# Reconstruction of a Pediatric Distal Phalanx Amputation With Stacked Integra Dermal Substitute: A Case Report

**DOI:** 10.7759/cureus.58856

**Published:** 2024-04-23

**Authors:** Naomi H Kelley, Tori L Shaver, Nathan T Morrell

**Affiliations:** 1 Orthopedics Hand Surgery, University of New Mexico Health Sciences Center, Albuquerque, USA

**Keywords:** composite tissue allotransplantation, dermal substitute, trauma, amputations, hand, digits, pediatric, surgery

## Abstract

Finger amputations in children present unique challenges and require special considerations compared to their adult counterparts. Maximizing length and preserving fingertip bulk and sensation is essential for maintaining a functional digit. Synthetic dermal substitutes have been recently used for soft tissue coverage for pediatric syndactyly as well as burn injuries; however, the literature discussing pediatric amputation cases with soft tissue damage proximal to the bony level is limited.In this case, we report a two-year-old patient who developed dry gangrene of her right index finger after multiple rabbit bites and underwent an amputation through the distal interphalangeal joint. Circumferential soft-tissue debridement proximal to the tip of the middle phalanx was required, leaving substantial exposed bone with no soft tissue envelope. We report our experience of single-stage stacking Integra dermal substitute directly onto the exposed bone to provide both finger bulk and soft tissue coverage.The patient displayed no functional limitations three years post-surgery.For instances when local or distant flap coverage may not be feasible, we present a novel technique to reconstruct, provide bulk, and preserve length in pediatric finger amputations. This case highlights that the utility of dermal substitutes is expanding and are providing more technical options.

## Introduction

Children under five years of age sustain finger amputations at a rate of 15.3/100,000, the highest among all age groups [1].​ The most common injury is a crush injury of the distal phalanx [2]. In a retrospective analysis of 245 patients, only 2% of pediatric amputations were secondary to animal bites or scratches [2]. To date, there are no articles that describe the management of a gangrenous digit after an animal bite in a child.

When considering pediatric finger amputations, there are several unique challenges and considerations in order to preserve as much length and function as possible. In terms of anatomy, a through-joint amputation (i.e., disarticulation) is preferred rather than a through-bone amputation due to the potential for bony overgrowth in children when the amputation is through bone. O’Neal et al. [3] report that an amputation through the diaphysis required revision for overgrowth in 45% of their amputations.

When needed, soft tissue coverage after pediatric finger amputation may be particularly challenging. Although there are limited studies that address pediatric amputations requiring soft tissue coverage specifically, there is extensive literature that addresses soft tissue coverage for congenital syndactyly, avulsion injuries, and burns [4]. Traditionally, syndactyly and burn injuries are repaired with skin grafts. However, recent advancements in synthetic dermal substitutes such as Integra Dermal Regenerative Template (Integra, Plainsboro, NJ), Hyalomatrix Hyaluronic Acid Wound Device (Anika, Bedford, MA), and MatriDerm (MedSkin, Billerbeck, Germany) have shown similar outcomes [5-7]. Given this, dermal substitutes have become an increasingly popular option for children with complex soft-tissue defects.

We present a case of a two-year-old patient who underwent a right index finger distal phalanx amputation with circumferential soft-tissue debridement proximal to the tip of the middle phalanx. In this case, we report our experience of single-stage stacking Integra dermal substitute directly onto exposed bone and cartilage to provide both finger bulk and soft tissue coverage. The patient’s parent was informed and consented to the inclusion and publication of this article.

## Case presentation

**Figure 1:**
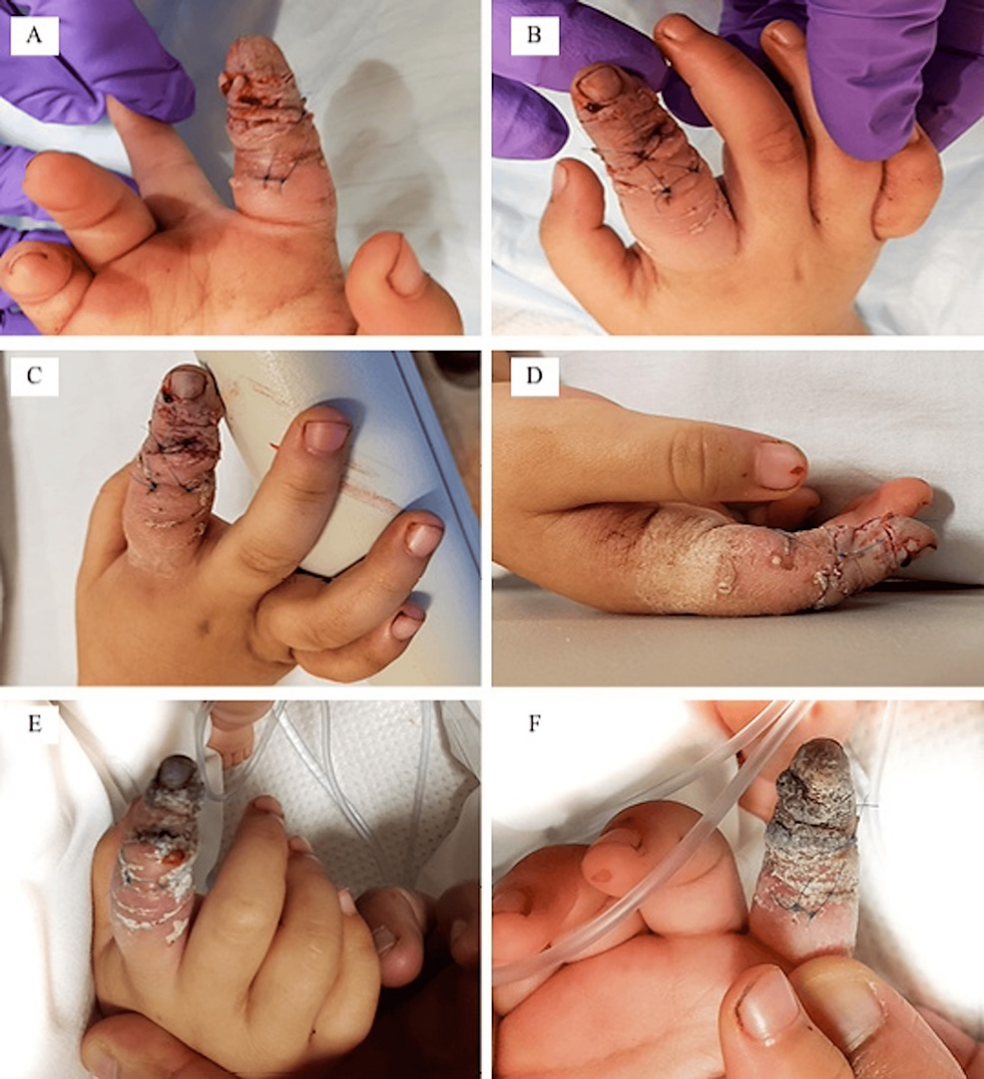
Pre-operative photos taken upon hospital admission. (A, B) Pre-operative photos taken upon hospital admission approximately one day after the initial injury. (C, D) Pre-operative photos taken approximately two days after injury. (E, F) Pre-operative photos taken approximately four days from injury demonstrating progression of dry gangrene of the index finger.

An otherwise healthy two-year-old girl sustained multiple rabbit bites to her right index finger. She was evaluated at two other hospitals prior to her presentation at our institution one day after her initial injury (Figures 1A, 1B). The patient was admitted to the hospital for antibiotics and topical vasodilator treatment. Over the subsequent days, she developed cellulitis and ischemic changes to the tip of her index finger (Figures 1C, 1D). Despite attempts to prevent vascular compromise, the fingertip developed dry gangrene four days after her initial injury (Figures 1E, 1F). At this point, amputation was discussed with her parents, who expressed interest in maintaining the maximal length possible.

**Figure 2:**
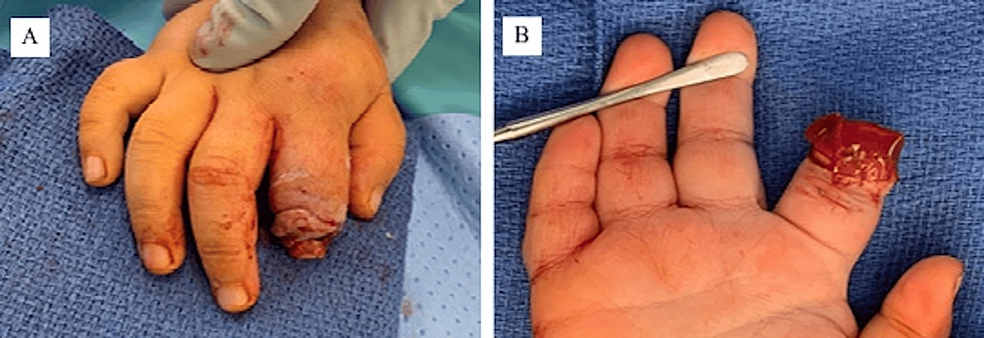
Intra-operative photographs demonstrating the disarticulated distal phalanx and circumferential soft tissue debridement until adequate bleeding tissue was reached. (A) The distal half of the middle phalanx extends beyond the soft tissue envelope. (B) The index finger after placement of the stacked Integra dermal substitute.

The patient underwent right index finger amputation. A distal interphalangeal joint disarticulation was performed, however the soft tissue required debridement to the middle of the middle phalanx due to soft tissue necrosis. The nonviable skin and soft tissue were debrided circumferentially until adequate bleeding was present at the margin (Figure 2A). This resulted in the distal half of the middle phalanx being without soft tissue coverage (i.e., the distal half of the middle phalanx significantly protruded beyond the end of the soft tissue envelope).

At this point, a variety of local advancement flap options were considered; however, due to the remaining damage from the animal bites, the reliability of these local flaps was considered compromised. Crossed-finger flap was considered though a single-stage technique was preferred. While more distant coverage options existed, ultimately the decision was made to utilize Integra dermal substitute for fingertip reconstruction and soft tissue coverage. Rather than place a single bilayer template, the inner layer of the Integra template was removed from the silicone outer layer and stacked over the bone such that there were two or three layers of the inner matrix beneath the silicone. This created a cap over the exposed bone with a similar bulk to the remaining finger (Figure 2B). The Integra template was secured in place with a 5-0 Monocryl suture. The wound was dressed with sterile Xeroform, gauze, and Kling. Finally, the patient was placed in a long arm univalved cast.

**Figure 3:**
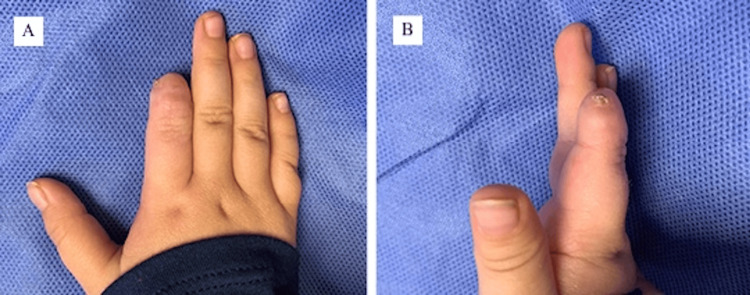
(A, B) Patient achieved complete epithelialization without bony protrusion of the index finger at approximately three months post-operatively.

The patient’s wound was evaluated three weeks post-operatively which demonstrated adequate vascularization without areas of ischemia or progressive necrosis. After discussion with the patient’s parents, the decision was made to allow the fingertip to epithelialize by secondary intention rather than place a skin graft. At one month post-operatively, there was evidence of skin epithelialization over the radial and ulnar aspects of the stump with local granulation tissue at the tip of the digit. The silicone layer was removed at this point. By three months post-operatively, the right index finger was completely epithelialized (Figures 3A, 3B).

**Figure 4:**
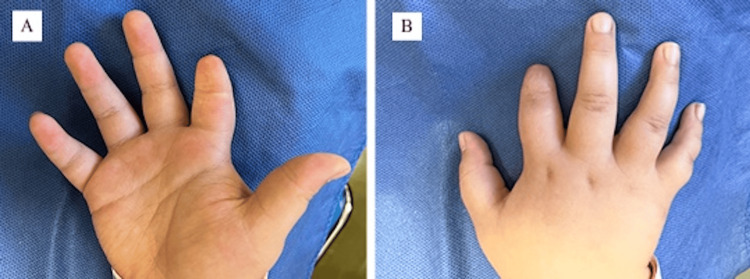
(A, B) Patient at three-year follow-up with appropriate skin coverage.

To date, the patient is now three years after her procedure and continues to demonstrate appropriate skin coverage without bony protrusion (Figures 4A, 4B). The patient’s parents have trialed a silicone oppositional finger prosthesis for cosmetic reasons, though the patient has shown no obvious complications or functional deficits following the index finger distal phalanx amputation.

## Discussion

Finger amputations are rare among children but may lead to significant complications and disability in cosmesis, sensation, and long-term function of the digit and hand. This case study reports a two-year-old patient with necrosis of the right index finger distal phalanx following animal bites and highlights a novel soft-tissue reconstruction technique using a single-staged, stacked Integra dermal substitute. Although there is growing data in support of dermal substitute use for burns and pediatric syndactyly reconstruction, the literature remains sparse regarding its use for soft tissue coverage for finger amputations and direct application onto bone and cartilage.

In the pediatric population, the need for soft-tissue coverage on digits most commonly arises in the setting of traumatic finger amputations secondary to crush injuries [8]. Regardless of the mechanism, the goal after any finger amputation is to preserve as much bone and finger length as possible, minimize the risk of infection, have a durable soft tissue envelope, and maximize function. Several soft-tissue reconstruction techniques are effective for fingertips including V-Y advancement flaps [4,9], cross-finger flaps [9,10], thenar flaps [11], and homodigital neurovascular island flaps [12].

While each of these techniques has specific benefits and indications, our patient had a unique injury that precluded or made the use of these methods less desirable. For example, due to the nature of having sustained many small bites throughout the hand, the proximal tissue over the index finger was not reliable for V-Y advancement or reverse homodigital neurovascular island flaps. Additionally, the patient required soft tissue debridement proximal to the distal aspect of the middle phalanx leaving bone protruding beyond the wound edge without soft tissue coverage. While children have excellent regenerative potential, given that more than half of the bone was circumferentially protruding from the wound, there was concern that it would potentially not heal by secondary intention. The prolonged amount of time required to achieve epithelialization in this case corroborates this concern. Lastly, considering the patient’s age, having to undergo multiple procedures as required for a cross-finger flap or thenar flap was deemed less ideal. Altogether, these factors created a difficult challenge that required a creative soft-tissue solution.

In light of these challenges, the Integra dermal substitute was stacked and applied directly onto bone and cartilage to build up the remaining fingertip in an attempt to preserve the middle phalanx. Preserving the flexor digitorum superficialis tendon insertion on the middle phalanx was considered important to preserve functional flexion of the remaining index finger. Soft tissue coverage was needed to avoid a more proximal amputation through the proximal interphalangeal joint. Integra is a bilayer of inner collagen matrix and outer silicone that serves as a scaffold for dermal regeneration [13]. In our case, the collagen matrix was stacked to improve the soft tissue bulk. The silicone layer was retained as the outermost layer as the wound was allowed to heal by secondary intention, without proceeding with secondary skin graft coverage after the initial stage. Although there is a growing amount of literature discussing Integra’s use in syndactyly reconstruction [13], burns [14], and diabetic foot reconstruction [15], stacking integra directly onto exposed bone is rarely reported in the literature, particularly in the pediatric population.

There are several studies that have described the successful application of Integra directly onto tendons and bone. Dantzer et al. [14] reported a series of 15 hands with severe burns in which one patient required a finger amputation that was then successfully grafted with Integra over exposed bone and soft tissue. Similarly, Clerici et al. [15] reported a series of cases in which Integra was applied directly over bone and exposed tendons after amputation and extensive debridement of diabetic foot infections. In contrast to our patient, the amputated extremities in these cases had intact viable surrounding soft tissue despite bone and tendon exposure. Thus, Integra was used in both of these cases as a single dermal regeneration template that was later skin grafted. Most similar to our case, Jacoby et al. [16] reported successful soft tissue coverage using one-stage integra onto the bone for fingertip amputations; however, the bone was flush with the soft tissue level in all of their patients, not protruding as in ours.

In regard to stacking Integra, there is scarce literature describing this technique. Jeng et al. [17] reported a case in which a patient sustained a pit viper bite to the right index finger that required urgent debridement of approximately 60% of the dorsal aspect of the finger leaving exposed tendon and bone. In this case, Integra was initially applied as a single layer and allowed to mature before the silicone layer was removed and additional layers of Integra were stacked over the wound. Similarly, Carothers et al. [18] reported a case during which Integra was used to fill a large soft tissue defect for a tumor resection in the palm. In this case, the patient underwent an initial monolayer covering of Integra and subsequently elected to have Integra layering performed three weeks after the index procedure. In both of these examples, Integra was initially applied as a monolayer that later underwent additional stacking of the dermal substitute. In contrast to these cases, we stacked the Integra circumferentially around the exposed bone of the amputated finger during the index procedure to avoid additional surgeries and allowed for healing by secondary intention. At approximately three weeks post-operatively, our patient displayed adequate take of the dermal substitute without signs of infection or failure.

Although Integra was successfully used in our case, there are several synthetic dermal substitutes that have demonstrated promising results in similar applications. For example, Hyalomatrix is a hyaluronic acid scaffold on a silicone membrane that has shown success in congenital syndactyly release and reconstruction [7]. Similarly, Matriderm, an elastin and collagen matrix, has been utilized for extensive full-thickness skin defects in the wrist and hand in the setting of burns and trauma [19]. Both Hyalomatrix and Matriderm have shown success for soft tissue coverage in traumatic finger amputations [20]. (Poster: Dedo D, Pirkle S, Prabhakar P, et al. Use of Hyalomatrix In Pediatric Post-Traumatic Fingertip Amputation Tissue Loss. American Society for Surgery of the Hand Annual Meeting; October 5-7, 2023.)

## Conclusions

Pediatric finger amputations are rare but potentially debilitating injuries. The use of dermal substitutes such as Integra have shown utility in complex soft tissue coverage for congenital syndactyly, burn, and avulsion injuries in children. This case demonstrates a novel technique of single-stage stacking integra directly over bone and cartilage to reconstruct, provide bulk, and preserve length for an index finger distal phalanx amputation. Due to the amount of bone exposed, healing by secondary intention or by occlusive dressing alone was unlikely to be successful. Our patient healed well and continues to demonstrate no significant complications or functional limitations in her amputated digit after a three-year follow-up period. Ultimately, the flexibility of dermal substitutes for soft tissue reconstruction may continue to find new applications beyond what is presented here.
